# Washing of the Stomach — Remarkable Effects of the Internal Use of Carbolic Acid in the Treatment of Fevers—Use of Phosphate of Lime in the Treatment of Chronic Bronchitis

**Published:** 1882-04

**Authors:** 


### Original Translations.

ARTICLE X.

WASHING OF THE STOMACH.—REMARKABLE EFFECTS OF THE INTERNAL USE OF CARBOLIC ACID IN THE TREATMENT OF FEVERS. Translated for the CHICAGO MEDICAL JOURNAL AND EXAMINER. By AUSTIN C. RAMSBY, Chicago.

*Washing of the Stomach.*—With the perfection and simplicity of the instrumental outfit, the washing of the stomach is going to take an important part in the practice of medicine. It constitutes a precious resource against affections of the stomach, and all the physicians who have tried it unanimously report happy results.

The instruments for that operation are all well known. The pump of Kusslmaul, the Dujardin-Beaumetz instrument, the Siphon-tube of Fancher, have all been presented to the readers of the *Revue Médicale.* Dr. Fancher's instrument seems to be the best, for two reasons: 1st. The facility of its introduction, which even the patient can do himself after a few operations. 2d. Because most everywhere it can be improvised—for a piece of rubber tubing of 1.50 long and 10 to 12 millimeters in diameter can almost always be had, and every household has a common funnel which can be used. Before speaking of the therapeutical indications for the washing of the stomach, we will describe the operation:

The patient should be sitting, the head looking directly forward, at almost a right angle with the vertical column. Some physicians prefer reversing the head backward, in order to introduce the sound vertically.

For pusillanimous patients the dorsal becubitus might offer certain advantages. In the first operation, to establish the habit and overcome the spasms of the pharynx, the use of the rigid sound is preferable; the common œsophagial sound answers the purpose well. But when, after repeating the operation a few times the patient knows how to introduce the rigid sound without pushing it, but by the simple action of deglutition, then the common red rubber tube must be substituted for the sound. The tube must be well oiled. The tube for females ought to be of smaller caliber than for males. A mark on the tube at 45 to 50 centimeters from its free extremity will indicate, when touching the lips, that the stomach has been reached. But this generally is known by a spasm of the cardia, which react to a slight pressure and causes an effort to vomit. Sometimes, but rarely, there is a sudden escape of gases, and of the contents of the stomach; this must be borne in mind in order to protect the patient's clothing. To fill the tube the funnel must be raised one meter above the level of the stomach, and filled with liquid to the amount of from one to two liters; then, by bringing it below the epigastric region, the fluid injected can be seen filling the funnel and holding in suspension the matter contained in the stomach. Dr. Constantin Paul advises the use of cold alkaline water, terminating the operation with warm water. The antiseptics, etc., are highly spoken of.

The operation terminates with the introduction of from 2 to 300 grams of milk in the stomach, and, to prevent the pyloric dilatation, the abdomen must be compressed with a large bandage, or, when possible, have the patient walk for a few minutes.

Such are the details of the operation according to Dr. Paul. We will see now what benefits we can derive from its use.

The washing of the stomach will instantly suppress the pains in cases of neuralgia and chronic gastritis; it will calm the consecutive oppression in cases of dilatation of the stomach.

It stimulates the appetite, and brings a rapid augmentation in weight. Drs. Sée, Buocquoy, Ferrand and Paul, have had surprising results from it in cases of gastritis, hysterical vomiting, dyspepsia, ulcers and cancers of the stomach; in this last, it is true, the relief is only temporary. Dr. Paul has even utilized it in a case of strangulated hernia, to save the patient the disagreeableness of fœcal vomiting.

Its use in cases of recent poisoning need not be commented upon—*Revue Médicale*..

---

REMARKABLE THERAPEUTICAL EFFECTS OF THE INTERNAL USE OF CARBOLIC ACID IN THE TREATMENT OF PYREXIAS. By DR. RAYMOND.

It is in the course of typhoid fever, particularly, that Dr. Raymond has obtained happy results from the use of this medical agent, given both in the form of pills and lavements.

Dr. Raymond stated before the Biological Society, that instead of having recourse to the old therapeutical doses of 10 to 12 grams, he prescribed it at the rate of one gram per day, 50 centigrams in three pills, and 50 centigrams in lavement.

Its effects are manifested soon after the taking. Lowering of temperature of from 3 to 4 Centigrade, and a considerable diaphoresis take place, which in a half an hour become intense.

Is this diaphoresis caused by carbolic acid? Dr. Raymond has plainly demonstrated that it is; by injecting ¼ of a milligram of duboisia hypodermically he has suddenly stopped the diaphoresis, the lowering of temperature taking place as before. The results are not always identical, being more marked after two or three days of treatment, when it becomes more rapid and energetic in its effects.

Such splendid success made him bold. He raised the dose from one to two grams, given as in the first dose, and he noticed signs of poisoning, dropping of temperature to 34°, convulsions, darkening of the urine. How can we justify, in the presence of such facts, the doses of 10 to 15 grams, which we used to administer in the past?

Dr. Raymond tried to use the carbolate of sodium for carbolic acid, giving that salt at the rate of 1 gram in 50, with the precaution that, according to Hallopean, the patient must be kept under the constant influence of the drug. The same happy results were obtained, with the addition of this: that it never exposes the patient to the poisonous effects of pure carbolic acid mentioned above. Carbolic acid is a very efficient agent in the treatment of contagious fevers, but its action is merely to reduce the temperature; in typhoid, for instance, it does not modify in any way the development of the different stages of the disease.

Given in form of lavements of 50 centigrams, and topically with the solution at 1-50, it is said to give excellent results in erysipelas.

In an operation for empyema, there was injected, by mistake, into the pleural cavity one liter of carbolic acid solution of 1.20; there was a sudden dropping of the temperature to 34°, and for nine days the urine of the patient was of dark color, notwithstanding the use of the sulphate of sodium. A fact worthy of notice is, that in tuberculosis, a non-contagious disease, carbolic acid has no influence over the fever.

As to the physiological mode of action of this agent, particularly in typhoid: It is not, according to Dr. Raymond, in its influence of immediate contact with the seat of the lesions that it must be looked for, because the carbolic acid is decomposed long before it reaches the lower part of the alimentary canal. The fall in the temperature is in this case of nervous origin, resulting from an action of carbolic acid on the great cells of the anterior cornua of the cord, produced through the medium of the vascular system, and, in the meantime, while this is manifested by a fall of temperature, it affects the whole of the sudorific gland. The appearance of convulsions, when too large a dose has been taken, is another point in favor of this hypothesis.—*Le Medicin Practicien*, July 30, 1881.

---

ON THE USE OF PHOSPHATE OF LIME IN THE TREATMENT OF CHRONIC BRONCHITIS. By DR. V. BRIDALLET. (*Revue Médicale, Feb.* 25, 1882.)

Phosphates, and particularly phosphate of lime, are so often used in the practice of medicine that I think it my duty to report a very remarkable recovery brought on by them.

In August, 1879, I was consulted by a young man, æt. 18, suffering from what had been pronounced by other physicians chronic bronchitis. A very careful examination confirmed the above.

Panting on the least exercise, violent and repeated coughing, more severe in the morning, abundant expectorations, loss of appetite and vomiting, the patient was very weak, extremely lean and complained of violent headaches. Percussion revealed nothing peculiar. On auscultation, rough breathing in the infra-clavicular region and under the scapula behind ; over the infra-mammary region very marked sibilant râles covering the vascular murmur, pulse weak and frequent, temperature below normal.

The above symptoms pointed to a more serious state of affairs than is generally met with in the average case. Here we had chronic bronchitis, complicated with a very advanced stage of cachexia.

I analyzed the urine of 24 hours; the result was : Urea, 11 grams ; uric acid, appreciable ; sugar, traces ; phosphate of lime, 5 grams—50.

The treatment had been rational up to that time : wine of cinchona, cod-liver oil, tar, soothing syrups. The same was continued, adding the milk diet, about two liters per day.

In November no change for the better had taken place. A second analysis of the urine revealed: Urea, 14 grams; uric acid, 45 centigrams ; sugar, traces ; phosphate of lime, 6 grams—40.

The patient was excreting an excess of phosphates. The results obtained from the treatment were insignificant. The urea had increased but the patient was losing his phosphate of lime, and the headaches were more severe. In May of the next year I saw him ; he was entirely discouraged. I continued the milk and cod-liver oil, but in doses of one tablespoonful a day, adding the phosphoric treatment, giving Reinvillier's syrup of phosphate of lime, one teaspoonful three times a day (equivalent to 9 grams), and daily frictions with phosphorated oil. I prescribed a trip to the country.

After three months of country air, of Reinvillier's syrup, of frictions with phosphorated oil, I saw the patient; the cough had diminished considerably, the headaches were less severe and less frequent, and the patient could indulge in long-continued outdoor exercise. The same treatment was continued, and in January, 1881, the cough and headache had disappeared, ascultation revealed a normal respiration and sounds, the expectoration was insignificant, the appetite was normal. A third analysis of the urine revealed no traces of sugar, urea and uric acid in normal proportions, and the enormous loss of phosphate of lime had stopped. I discontinued the milk, cod-liver oil and the frictions, but to assure a complete recovery, I continued Reinvillier's syrup for three months at the spoonful a day.

I saw the patient since and he is in perfect health.

This is another proof of the great value of phosphate of lime as a reconstituent, and of the significance of dephosphatization in most chronic diseases.

---

THE NIGHT MEDICAL SERVICE.—Brooklyn is just about to put into effect within her limits the statute in regard to the organization of a night medical service, such as has now been in operation in this city for one year. This service is in every respect a good thing—an important and almost indispensable contribution to the organization of the night life of a city. As the official report for the year recently made here showed, it has kept, as to expenditure, well within the limits of the very reasonable appropriation made; it has enabled a number of persons taken suddenly ill with distressing maladies to obtain immediately skillful medical attendance, and it has proved a welcome provision to the doctors who were called, as well as to those who, through this law, were freed from the common importunity to which doctors in cities are nearly always exposed, to go out at night and attend sick persons whom they do not know, and who cannot pay a fee. In every city, things would be better for the organization of such a service.—*Medical Gazette.*

